# Posterior Tracheal Wall Laceration Following Tracheostomy: A Progressive Nightmare Under Long-Term Ventilation, Requiring Complex Repair With vvECMO Support—A Case Report

**DOI:** 10.1155/crcc/6643639

**Published:** 2025-05-28

**Authors:** Stefan Welter, Dietrich Stockhausen, Dany Balke, Varun Gupta, Wojciech Dudek

**Affiliations:** ^1^Department of Thoracic Surgery, Lungenklinik Hemer, Hemer, Germany; ^2^Department of Thoracic Surgery, Marienkrankenhaus Soest, Soest, Germany

**Keywords:** subglottic stenosis, tracheal membrane laceration (TML), tracheal reconstruction, tracheostomy, vvECMO

## Abstract

Untreated tracheal membrane laceration (TML) may have life-threatening consequences. We present a case of untreated TML during or after tracheostomy. Air leakage along the cannula after tracheostomy was treated with raising cuff pressure up to > 100 mmHg and enlarging the tracheal lumen in the area of TML. Finally, small movements of the neck led to immediate blockade of the tubetip and repeated life-threatening asphyxia. Immobilization, anxiety states, addiction to sedatives, and several situations with hypercapnic coma led to ICU transferal to our tertiary thoracic center. Chronic TML was diagnosed with flexible bronchoscopy through the larynx and the tracheostomy. Operative repair under veno-venous extracorporeal membrane oxygenation (vvECMO) required tracheal transection at the lower border of the tracheostomy and detachment of both edges of the ruptured tracheal membrane from the anterior vertebral ligament and reconstruction with a running suture. With a 3/4 reanastomosis of the trachea, a new tracheostomy channel was created. Within the following 4 months, no further ventilation problems occurred. We conclude that untreated TML after tracheostomy may develop in a vicious circle with a permanent risk of death under long-term ventilation. Late repair can be complex even in experienced hands.

**Summary**

• Tracheal membrane laceration (TML) during tracheostomy may remain unnoticed.
• Increasing cuff pressure to stop air leakage leads to increasing trachea diameter.
• Greater mobility of the cannula can create a false passage into the mediastinum.
• Repeated blockade of the ventilation chain may lead to asphyxia and panic attacks.
• Treatment of chronic TML may require veno-venous extracorporeal membrane oxygenation (vvECMO) for open reconstruction.

## 1. Introduction

Iatrogenic TML has been extensively described in terms of its acute management, particularly as a complication of oral intubation. The therapeutic approach is contingent on the length of the lesion and the depth of the posterior wall injury. A variety of therapies have been proposed, ranging from conservative treatment, fibrin glue application, endotracheal suture, and transtracheal minimally invasive reconstruction to open posterior wall suture via right thoracotomy or stent placement [1]. The commonality between these conditions is the management of acute complications, including subcutaneous emphysema, pneumothorax, ventilation issues, and hemoptysis [2]. Drawing upon our expertise in the field, it is asserted that the acute phase of problems can be surmounted within a few days. There is a paucity of literature on the long-term consequences, including mediastinal or endotracheal herniation, lumen closure during coughing, and instability of the posterior tracheal wall. However, this problem is well documented in major thoracic surgery and pneumology centers [3]. It is evident that tracheal stenosis and tracheomalacia are more prevalent long-term complications arising from tracheostomy tube placement [4]. Iatrogenic TML during percutaneous dilatational tracheostomy, or in rare cases during surgical tracheostomy, is a problem that has gone largely unnoticed to date. The estimated risk for tracheobronchial injury during percutaneous dilatational tracheostomy is one in 575 [5]. However, the complication rate associated with percutaneous dilation tracheostomy can reach up to 16% in obese patients [6]. Tracheostomy tube dislocations have been documented in up to 2.6% of cases, and the acute event is associated with a mortality rate ranging from 25% to 100% [7]. The present case report details a patient with a combination of nearly all possible late complications of tracheal posterior wall injury during or after a tracheostomy, which resulted in a life-threatening permanent condition. It was only possible to successfully address the serious threat to life by performing a complex surgical reconstruction.

## 2. Case Presentation

**Figure 1:**
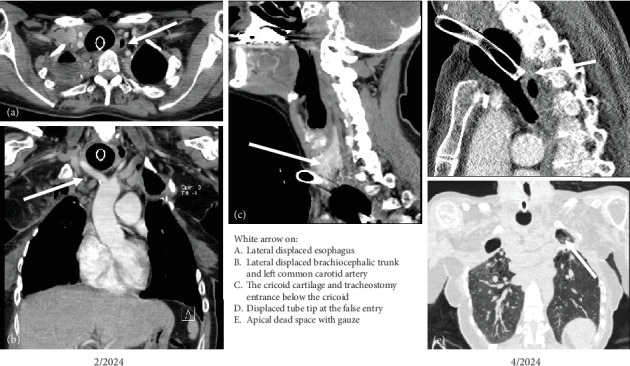
Contrast-enhanced CT scan of the chest before tracheal reconstruction. Legend 1: The image displays the chest CT scan prior to the operative reconstruction. As illustrated in Figure 1, the white arrow denotes the (a) lateralized esophagus, (b) the distended trachea with displacement of the brachiocephalic artery, (c) the cannula's entry at the level of the cricoid, (d) the posterior contact of the cannula's tip with the TML and the false passage, and (e) the bilateral apical spaces on the remaining lungs.

In April 2024, a 43-year-old female patient was transferred to our institution due to recurrent asphyxia under long-term ventilation. For a period exceeding 4 years, she was mechanically ventilated via tracheostomy in a respiratory care unit designated for long-term mechanically ventilated patients (IChome). The tracheostomy was performed in 2019 as a permanent surgical tracheal access, involving the open division of the thyroid isthmus and the creation of a tracheal opening between the third and fourth tracheal rings. The patient's medical history includes Type 1 diabetes, tobacco-induced emphysema, and polyangiitis. Since 2010, she has undergone repeated surgical procedures on both sides due to the following respiratory conditions: spontaneous pneumothorax, necrotizing lung abscess, aspergilloma, empyema, hemoptysis, and persistent air leakage. The sequence of surgical interventions involved the removal of the right upper lobe, followed by Segment 6 of the middle lobe, and subsequently the left upper lobe. The persistent air leakage and B6-insufficiency on the right side were treated with a latissimus muscle flap coverage. Bilateral axillary thoracostomies were performed in 2019 to address the presence of bilateral empyemas. In early April 2024, bilateral subclavicular thoracostomies were deemed necessary due to the obstruction of the axillary openings by scar tissue and granulations. It is evident that, over the course of time, the patient developed an irreversible hypoxic respiratory insufficiency. Since March 2024, there have been repeated occurrences of sudden obstruction of the ventilation chain, resulting in severe hypoxemia, due to mediastinal dislocation of the tube tip (Figure 1).

The patient repeatedly inflated the tube-cuff to 120 mmHg, with the intention of preventing air leakage along the cannula and averting another dislocation. The treating staff reported the rupture of the cuff on multiple occasions per week, necessitating an emergency change of cannula.

**Figure 2:**
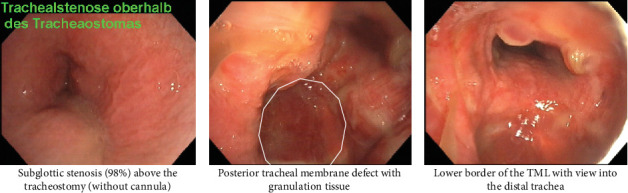
Flexible bronchoscopy 1 week before operative repair. Legend 2: Bronchoscopy pictures demonstrating (a) the subglottic stenosis from the laryngeal view, (b) the laceration covered with granulation tissue and marked with the white ring, and (c) the distal trachea with the lower end of the TML.

Upon admission, a substantial longitudinal and oval-shaped widening of the tracheostomy aperture was observed. The patient exhibited severe cutaneous inflammation, accompanied by partial ulceration, resulting from persistent leakage of purulent bronchial secretions. In the case of known subglottic stenosis (98%), a tracheostomy speaking valve had not been used for a considerable period. In this case, the 9.5 mm tracheostomy cannula (Rüsch Tracheoflex Extra 9.5 mm, Teleflex Medical GmbH, Germany) was utilized, with the result that ventilation could be maintained in the optimum manner (see Figure 2).

The patient exhibited a pronounced response of severe panic attacks whenever the ventilator alarm sounded, indicating the presence of airway obstruction. Subsequently, the cannula was directed towards the larynx, thus facilitating ventilation once more. The cuff was rendered permanently ineffective by pressures in excess of 80 mmHg. It was hypothesized that, with reduced pressure, it would be impossible to prevent air leakage along the tracheostoma channel. Due to the potential for acute blockage of the cannula during movement, the patient categorically refused any mobilization, remained in a supine position, and demanded sedative medication at regular intervals. Following a series of hypoxic and hypercapnic comas, a transfer to a tertiary care chest hospital was initiated. Despite the patient receiving intensive care treatment for a severe respiratory infection, there were multiple occurrences of acute hypoxia and hypercapnia during the night. The patient was required to undergo bronchoscopic suctioning and repositioning of the cannula, in addition to undergoing short-term pure oxygen ventilation.

### 2.1. Diagnostic Work Up

A contrast-enhanced CT scan of the neck and thorax (Figure 1) showed a high tracheostomy entrance below the cricoid, a massively dilated upper and middle third of the trachea in the area of the cuff and almost direct contact of the cuff with the spine. Both internal carotid arteries were pushed to the sides. Flexible bronchoscopy showed an almost occluded subglottic region that could be passed with a flexible bronchoscope after a 30-s retraction of the tracheal cannula. Furthermore, an elongated 6 cm O-shaped tracheal posterior wall lesion covered by granulation tissue was diagnosed (Figure 2). At the distal end there was a granulated false passage in the posterior mediastinum. The tracheal cannula with insufflated cuff was completely mobile. The cuff acted like a ball, in which the cannula could be moved in all directions without resistance, so that the tip of the cannula could be displaced ventrally against the cartilage rings, dorsally into the blind sac, or even against the right or left side.

### 2.2. Therapeutic Intervention

Surgical treatment was discussed and planned on an interdisciplinary basis with pulmonology, thoracic surgery, anesthesia, and the patient. The situation was assessed as follows:

1. There is a permanent risk of airway obstruction with potentially lethal consequences. A transfer away from the intensive care unit with complete monitoring is not possible under these conditions.
2. Due to the low tidal volume and the damage of the remaining lung, permanent ventilation with high inspiratory ventilation pressures is necessary. Jet ventilation during a surgical procedure will not be possible.
3. For the option of inserting a speaking cannula, airtight narrowing of the tracheostomy channel and, if possible, elimination of the subglottic stenosis is required.
4. Due to the massive adhesions of the mediastinum, large vessels, and lungs, circular mobilization and tracheal resection will not be possible or will be associated with a high risk.
5. Extracorporeal oxygenation is required to ensure oxygenation during the procedure with simultaneous free exposure of the surgical field.

### 2.3. Operation

**Figure 3:**
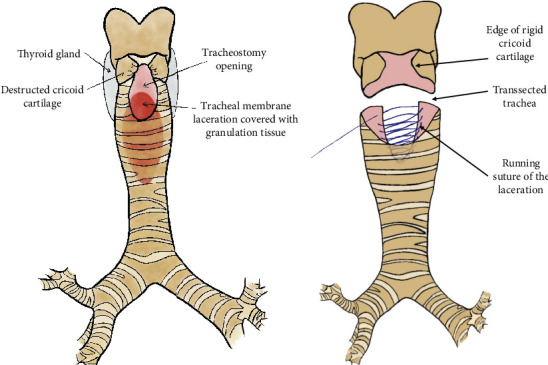
Illustration of the TML and the operative repair. Legend 3: Illustration of the TML with (a) the large tracheostomy opening and the destructed anterior cricoid and (b) the operative repair with the running suture of the posterior wall after transection of the trachea.

vvECMO with cannulation of the right jugular vein and right femoral vein was established and was able to provide oxygenation without ventilation with a blood flow of 4.5 L/min. The trachea was accessed via a collared incision with an inverted T to the cranial side to freshen the wound edges. After the exposure and mobilization of the straight neck muscles, the cricoid was found to be destroyed and pushed inwards in a U-shape. A first cartilage ring comprised of only soft granulation tissue. The thyroid isthmus was necrotically disintegrated, and both thyroid lobes were hardened by inflammation. Distally, the trachea was firmly adhered to the connective tissue around the brachiocephalic artery and vein. With the 5-mm-video camera, the situs was inspected deep inside the trachea. The posterior wall consisted of granulation tissue, and the spinal column could be palpated directly underneath with the finger (Figure 3a). The trachea was transected at the lower edge of the tracheostomy opening, and the posterior wall of the trachea was sharply dissected, exposing the esophagus and the lateral edges of the posterior tracheal wall. The granulation tissue was curetted from the anterior longitudinal ligament. The posterior membrane was then continuously sutured and adapted using a resorbable monofilament polyglycolic acid 3/0-suture (Maxon, Covidien, Medtronic, United States) (Figure 3b).

The anterior cricoid and first cartilage ring were elevated with multiple U-stiches through the hardened thyroid wall, so that an index finger could be passed through the former stenosis into the larynx. Then a 3/4 tracheal reanastomosis was performed, with continuous suturing of the posterior wall using 4/0 Maxon, and the lateral walls were sutured with single 3/0 Maxon stitches. This allowed significant narrowing of the tracheostomy opening. A flexible 8.5 mm tracheostomy cannula could be introduced, and with a low cuff pressure, the trachea was already airtight, with no air-leakage.

**Figure 4:**
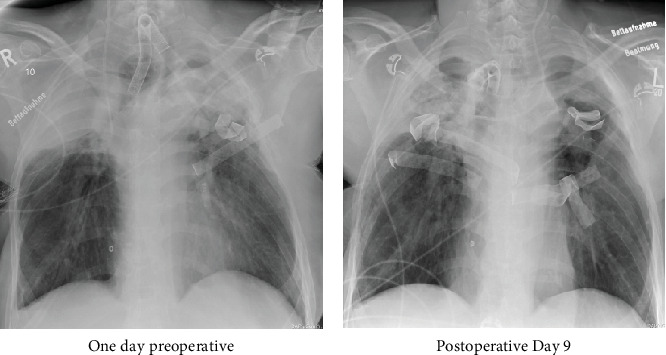
Pre- and postoperative chest x-ray. Legend 4: Chest x-ray on admission with (a) the spherical tracheal balloon of the tracheostomy cannula and (b) the tracheostomy cannula without any enlarged cuff and bilateral wound gauze in the apical thoracic spaces.

Wound healing progressed with minimal secretion around the cannula over the following 4 months. After a week, the flexible cannula was replaced by an 8.5 mm rigid speaking cannula with subglottic suction (Tracoe Twist Plus, TRACOE Medical GmbH, Germany) (Figure 4).

The accompanying bronchoscopy revealed a remaining 40% subglottic tracheal stenosis, an adapted posterior tracheal membrane with a small bulge near the anastomosis. The patient was transferred back into her IChome on postoperative Day 12. After 4 months, no dislocation of the cannula had occurred, and the tracheostomy channel was airtight with normal cuff pressure. The patient gave written consent to publish her case.

## 3. Discussion

Iatrogenic injury to the posterior wall of the trachea during tracheostomy placement is rare but may be life-threatening [7, 8] and may remain unnoticed during the procedure. The occurrence of late consequences has not been reported. The TML site is typically located opposite and below the stoma entrance. Acute tracheostomy tube occlusion is usually caused by mucous plug, obstructing granuloma, or the insertion of the tube in a false tract [9], as it occurred in our patient. Percutaneous dilatational tracheostomy can be associated with tracheal damage mainly caused by lateral puncture and rupture at the junction between cartilages and posterior wall. Immediate operative correction is necessary [10].

Patients who are selected for tracheostomy typically exhibit severe respiratory insufficiency and are unable to tolerate interruptions to their artificial ventilation. This case illustrates the possible long-term consequences of TML, implementing a detrimental cycle. Air leakage around the cannula was treated with increased cuff pressure.

The issue of air leakage around the cannula was addressed by increasing the cuff pressure. The cuff, which was nearly spherical in shape, expanded the defect, thereby allowing the cannula tip to shift in all directions, repeatedly dislocating into a mediastinal blind sac, which consequently led to repeated instances of hypoxic or hypercapnic coma. Fear, anxiety disorder, and panic dominated the patient's life. As a consequence of prolonged tracheostomy, repeated bilateral pleural empyemas, and aspergillomas, fibrotic changes occurred in the adjacent mediastinal structures, and large vessels became fixed to the anterior trachea. An autonomous descending fibrosis or necrotizing mediastinitis was not diagnosed during the course of the disease. Nevertheless, it appeared to be too hazardous to attempt the mobilization of the trachea for a resection and end-to-end anastomosis. It is evident that a complex tracheal reconstruction is the only procedure that has the potential to prevent the patient from developing a condition that could result in permanent life-threatening condition. vvECMO has been established as the optimal method to support complex interventions in the central airways [11]. The merits of the unobstructed surgical field and secure oxygenation in cases of severe pulmonary insufficiency have also been acknowledged by other authors [12]. The mortality rate is 0%, and the complication rate is minimal when the device is used during the perioperative period. The strength of this case report lies in the excellent visualization of the problem with diagnostic means and the comprehensive description of the pathophysiology, especially of the development of a life-threatening circle. However, the temporal parameters of the TML could not be delineated with precision. It is conceivable that the tracheal damage may have been occasioned by one of the tracheostomy tube changes.

## 4. Conclusions

Iatrogenic TML remains a severe and dangerous threat for the patient. In case of permanent tracheostomy, the inflated cuff prevents merging of the membrane and can lead to ongoing distention of the tracheal lumen and cause posterior wall instability. Operative repair is necessary, as early as possible, to prevent life threatening, long-term problems. The process of TML-repair in a state of chronic inflammation and scarring is complex and dangerous, but it is possible in experienced hands. The vvECMO is an excellent tool that facilitates a clear and unobstructed operative field.

## Data Availability

The data that support the findings of this study are available on request from the corresponding author. The data are not publicly available due to privacy or ethical restrictions. Data on this case are discharge letters, operation reports, and other personalized documentations that cannot be shared in public. Anonymized CT scans, bronchoscopy pictures, and chest x-rays are shared in this manuscript.
